# Radiosurgery-induced early changes in peritumoral tissue sodium concentration of brain metastases

**DOI:** 10.1371/journal.pone.0313199

**Published:** 2024-11-04

**Authors:** Arne Mathias Ruder, Sherif A. Mohamed, Michaela A. U. Hoesl, Eva Neumaier-Probst, Frank A. Giordano, Lothar Schad, Anne Adlung

**Affiliations:** 1 Department of Radiation Oncology, University Medical Centre Mannheim, Mannheim, Germany; 2 Department of Neuroradiology, University Medical Centre Mannheim, Mannheim, Germany; 3 Computer Assisted Clinical Medicine, Medical Faculty Mannheim, Heidelberg University, Mannheim, Germany; Goethe University Hospital Frankfurt, GERMANY

## Abstract

**Background:**

Stereotactic radiosurgery (SRS) is an effective therapy for brain metastases. Response is assessed with serial ^1^H magnetic resonance imaging (MRI). Early markers for response are desirable to allow for individualized treatment adaption. Previous studies indicated that radiotherapy might have impact on tissue sodium concentration. Thus, ^23^Na MRI could provide early quantification of response to SRS.

**Purpose:**

We investigated whether longitudinal detection of tissue sodium concentration alteration within brain metastases and their peritumoral tissue after SRS with ^23^Na MRI was feasible.

**Study type:**

Prospective.

**Population:**

Twelve patients with a total of 14 brain metastases from various primary tumors.

**Assessment:**

^23^Na MRI scans were acquired from patients 2 days before, 5 days after, and 40 days after SRS. Gross tumor volume (GTV) and healthy-appearing regions were manually segmented on the MPRAGE obtained 2 days before SRS, onto which all ^23^Na MR images were coregistered. Radiation isodose areas within the peritumoral tissue were calculated with the radiation planning system. Tissue sodium concentration before and after SRS within GTV, peritumoral tissue, and healthy-appearing regions as well as the routine follow-up with serial MRI were evaluated.

**Statistical tests:**

Results were compared using Student’s t-test and correlation was evaluated with Pearson’s correlation coefficient.

**Results:**

We found a positive correlation between the tissue sodium concentration within the peritumoral tissue and radiation dosage. Two patients showed local progression and a differing tissue sodium concentration evolution within GTV and the peritumoral tissue compared to mean tissue sodium concentration of the other patients. No significant tissue sodium concentration changes were observed within healthy-appearing regions.

**Conclusion:**

Tissue sodium concentration assessment within brain metastases and peritumoral tissue after SRS with ^23^Na MRI is feasible and might be able to quantify tissue response to radiation.

## Introduction

Stereotactic radiosurgery (SRS) is an effective localized therapy for patients with a limited number of brain metastases and SRS alone results in similar overall survival but with better neurocognitive outcomes than SRS and whole-brain radiotherapy (WBRT) [1–3]. With high precision and sharp dose gradients, SRS permits the application of ablative doses to brain metastases while sparing the surrounding peritumoral tissue. In the context of prolonged survival due to new systemic therapies, SRS can provide the possibility for repeated treatments of sequentially diagnosed brain metastases [4–6]. With extended survival, long term local tumor control gains additional importance. Thus, early markers of response to SRS would be desirable to allow physicians to individually adapt the prescription dose, the dose distribution (e. g., by applying a higher marginal dose or a higher central dose) or the fractionation scheme (e. g., by switching to a fractionated scheme that would allow for re-oxygenation) for (further) radiation treatments [7].

To date, response to SRS is assessed by serial magnetic resonance imaging (MRI), yet early evaluation of changes in size for response prediction has shown mixed results [8–11]. Various additional MRI techniques can be utilized to distinguish progression of brain metastases from pseudo-progression or radionecrosis [12–14].

With ^23^Na MRI derived sodium-weighted signal, changes of tissue sodium concentration (TSC), originating from changes in intracellular sodium concentration or extracellular volume fraction, can be detected [15–17]. While TSC has previously been shown to be elevated in malignant brain tumors compared to healthy brain tissue, treatment-induced changes of TSC in brain metastases or vestibular schwannoma after SRS have only recently been published [18–21]. As SRS affects brain metastases as well as the peritumoral tissue, ^23^Na MRI assessment of peritumoral tissue could provide early information regarding radiation dose effects and, together with TSC of brain metastases, could serve as marker for response to radiotherapy. We conducted a study to analyze the longitudinal changes of TSC in the peritumoral tissue of brain metastases, measured with ^23^Na MRI before and after SRS.

## Methods

A prospective study was conducted to investigate early tissue response to SRS. The study protocol was approved by the institutional review board (Ethikkommission II, Heidelberg University, Mannheim Medical Faculty, approval no. 2019–630 N-MA). Written informed consent was obtained from all patients. Written consent was checked and confirmed by the department’s clinical trials unit. In the recruitment period between 03 June 2019 and 29 May 2020, ^23^Na MRI scans were acquired from 12 patients with one or more previously untreated brain metastases stemming from a known primary tumor that were scheduled to be treated with SRS. For study inclusion, the volume of at least one brain metastasis was required to be ≥ 64 mm^3^ because of the nominal resolution of the ^23^Na MRI being 4x4x4 mm^3^.

### Data acquisition

^23^Na MRI scans were scheduled at three different time points:

- 2 days pre-SRS (baseline, T = SRS– 2 days): ^23^Na MRI protocol + ^1^H MRI routine protocol
- 5 days post-SRS (T = SRS + 5 days): ^23^Na MRI protocol
- 40 days post-SRS (T = SRS + 40 days): ^23^Na MRI protocol + ^1^H MRI routine protocol

Scan (I) and (III) were synchronized with routine ^1^H MRI acquisitions required for SRS planning or post-treatment observations. An additional appointment was scheduled for scan (II). After completion of scan (III), patients underwent follow-up with quarterly physical exams and routine MRI scans. Routine follow-up MRI scans were assessed according to RANO-BM criteria [8].

Data acquisition was performed with the 3T Magnetom Trio (Siemens Healthineers, Erlangen, Germany) and a dual-tuned transmit-receive 1Tx/1Rx ^1^H/^23^Na birdcage head coil (Rapid Biomedical, Rimpar, Germany). The MRI protocol included the ^23^Na MRI and a post-contrast ^1^H T1w MRI. The ^23^Na MRI was acquired after the administration of contrast agent, which has been shown to have no significant impact on the following TSC quantification [22]. A 3D radial density-adapted sequence was employed [23] using 9,000 spokes with 384 data points each, resulting in a measurement time of TA = 15 min. The relatively long repetition time of TR = 100 ms allowed minimization of T1-weighting effects and the short echo time of TE = 0.4 ms reduced T2*-weighting effects. Nominal image resolution was 4x4x4 mm^3^ but a zero-filling factor of 2 was introduced during image reconstruction, generating an apparent resolution of 2x2x2 mm^3^. Image reconstruction was performed in MATLAB 2018a (The MathWork Inc, Natick, MA, USA), using a regridding algorithm in k-space and a Hanning filter with the width of 4. The ^1^H routine MRI were acquired with a 12 channel ^1^H head coil, and the protocol included a T1w, T2w, and a contrast-enhanced (Dotarem, 0.2 ml/kg body weight) T1w 3D magnetization prepared rapid gradient-echo sequence (MPRAGE).

### SRS treatment

All patients were treated according to institutional standards with the Leksell Gamma Knife Icon (Elekta AB, Stockholm, Sweden). Segmentation, treatment planning and optimization was conducted with Leksell GammaPlan (Elekta AB, Stockholm, Sweden). Brain metastases were segmented using the contrast-enhanced MPRAGE image acquired at time point (I) as gross tumor volume (GTV). The planning target volume (PTV) was either identical to the GTV (frame fixation system) or an additional margin of 1 mm was added to the GTV to build the PTV (thermoplastic mask fixation). All patients were then treated with a single dose ranging from 16 Gy to 22 Gy prescribed to the 50% isodose line encompassing the PTV.

### TSC quantification

Additionally to the segmentation of brain metastases, two cylindrical regions (radius: 5 mm, height: 10 mm) within each patient’s healthy-appearing white matter (healthy ROIs, HR) were defined by a radiation oncologist. Isodose lines for each SRS were calculated with GammaPlan and transformed into DICOM structures. All ROIs were then exported from GammaPlan as DICOM RT files.

The acquired and reconstructed ^23^Na MRI were co-registered to the contrast-enhanced ^1^H MPRAGE, which had been used for SRS planning with the aim of being able to transfer the ROIs (GTV, isodose lines and HR) to the ^23^Na MRI. Therefore, all available ^23^Na MR images of each patient were co-registered to the same MPRAGE from scan (I). Co-registration was performed with the statistical parametric mapping software SPM12 (Wellcome Trust Centre for Neuroimaging, London, UK). Furthermore, automatic image segmentation into WM, GM, and CSF was performed for being able to exclusively evaluate WM and GM regions with no CSF impingement. Automatic image segmentation was also performed with SPM12.

Quantification was performed by normalization to the signal intensity (SI) within the patients’ left vitreous humor (VH). Three-dimensional ROIs were manually segmented within each patient’s MPRAGE, which was then transferable to all three ^23^Na MRI because of the image co-registration. The image’s SI was normalized based on SI within that ROI. TSC concentration within the VH was assumed to be constant at 145 mM, equivalent to the extra-cellular ^23^Na concentration [24, 25], and similar to the findings of Kokavec *et al*. [26]. A correction of the T1-weighting within the VH was introduced based on an estimated T1 relaxation time of the VH with T1(VH) = 50 ms as was previously suggested by Wenz *et al. [27]* because similar relaxation times were assumed in VH and CSF. The TSC-map was then calculated by:

$$TSC(Tissue)=SI(Tissue)\frac{145\;mM}{{SI}_{T1corr}(VH)}$$

DICOM RT structure files were imported into MATLAB with the Computational Environment for Radiological Research (CERR) [28]. It allowed to identify the irradiated and segmented regions from Leksell GammaPlan and enabled evaluation within MATLAB of those regions on the ^23^Na MRI.

TSC was measured within GTV, HR and the isodose areas of D = 2, 3, 4, 6, 8, 10, 12, and 18 Gy on all ^23^Na MRI scans of each patient. An isodose area was defined as the area enclosed by the corresponding isodose line with subtraction of the area enclosed by the preceding isodose line. The HR were evaluated to compare the TSC development within the irradiated regions to non-irradiated, healthy-appearing tissue of the same patient for each scan (I, II, and III). The different isodose areas were evaluated to investigate the effect of radiation on healthy tissue. Potential CSF regions within all ROIs were subtracted as described above.

### Statistical analysis

Mean TSC within brain metastases, HR, and within all isodose areas was compared between measurements at time points (I), (II), and (III). Differences were tested for statistical significance using the paired student t-test, and p < 0.05 was considered significant. The student t-test was applicable as the Kolmogorov-Smirnov test showed normal distribution.

The Pearson correlation test was used to evaluate a potential correlation between the radiation doses D and the mean TSC within the corresponding isodose area. If not otherwise indicated, values are reported with their mean ± standard deviation.

## Results

Twelve patients with a total of 14 brain metastases were evaluated. Mean dose of SRS was 21.4 ± 1.7 Gy and 2 patients (2 brain metastases) were treated with a frame fixation. Out of those, 9 patients (11 brain metastases) underwent all scheduled scans, 2 patients (2 brain metastases) only underwent scan (I) and (II) and one patient (1 brain metastasis) only underwent scan (I) successfully. Median follow up with serial MRI was 13.2 ± 8.4 months. Two patients showed local progression of treated brain metastases (defined as progressive lesion within or overlapping with the PTV). Patient characteristics including histology of the primary are presented in Table 1.

**Table 1:** Patients’ characteristics including the location of the evaluated metastases, metastases’ volume, radiation dose, primary histology, follow up characteristics and completed ^23^Na MRI. (BM: brain metastases).

Patient number	BM number	Location of BM	GTV (cm^3^)	Dose (Gy)	Primary histology	Progredient BM during follow-up MRI	Completion of scan (I)	Completion of scan (II)	Completion of scan (III)
1	1	Frontal left	0.423	22	NSCLC	no	yes	yes	yes
	2	Frontal left	0.315	22	NSCLC	no	yes	yes	yes
2	3	Frontal left	0.264	22	Mammary carcinoma	no	yes	yes	yes
	4	Frontal right	1.456	22	Mammary carcinoma	no	yes	yes	yes
3	5	Occipital right	0.124	16	NSCLC	no	yes	yes	no
4	6	Occipital right	1.637	22	NSCLC	no	yes	yes	yes
5	7	Occipital left	0.416	22	Malignant melanoma	no	yes	yes	yes
6	8	Postcentral right	0.411	22	Renal cell carcinoma	no	yes	yes	yes
7	9	Occipital right	0.723	22	Soft tissue sarcoma	yes (3.2 months after SRS)	yes	yes	yes
8	10	Cerebellar right	0.428	22	Esophageal cancer	no	yes	no	no
9	11	Frontal left	0.197	22	Renal cell carcinoma	no	yes	yes	no
10	12	Frontal right	0.891	22	CUP-syndrome	no	yes	yes	yes
11	13	Temporal left	1.075	22	Mammary carcinoma	yes (16.7 months after SRS)	yes	yes	yes
12	14	Occipital right	2.272	20	NSCLC	no	yes	yes	yes

**Fig 1:**
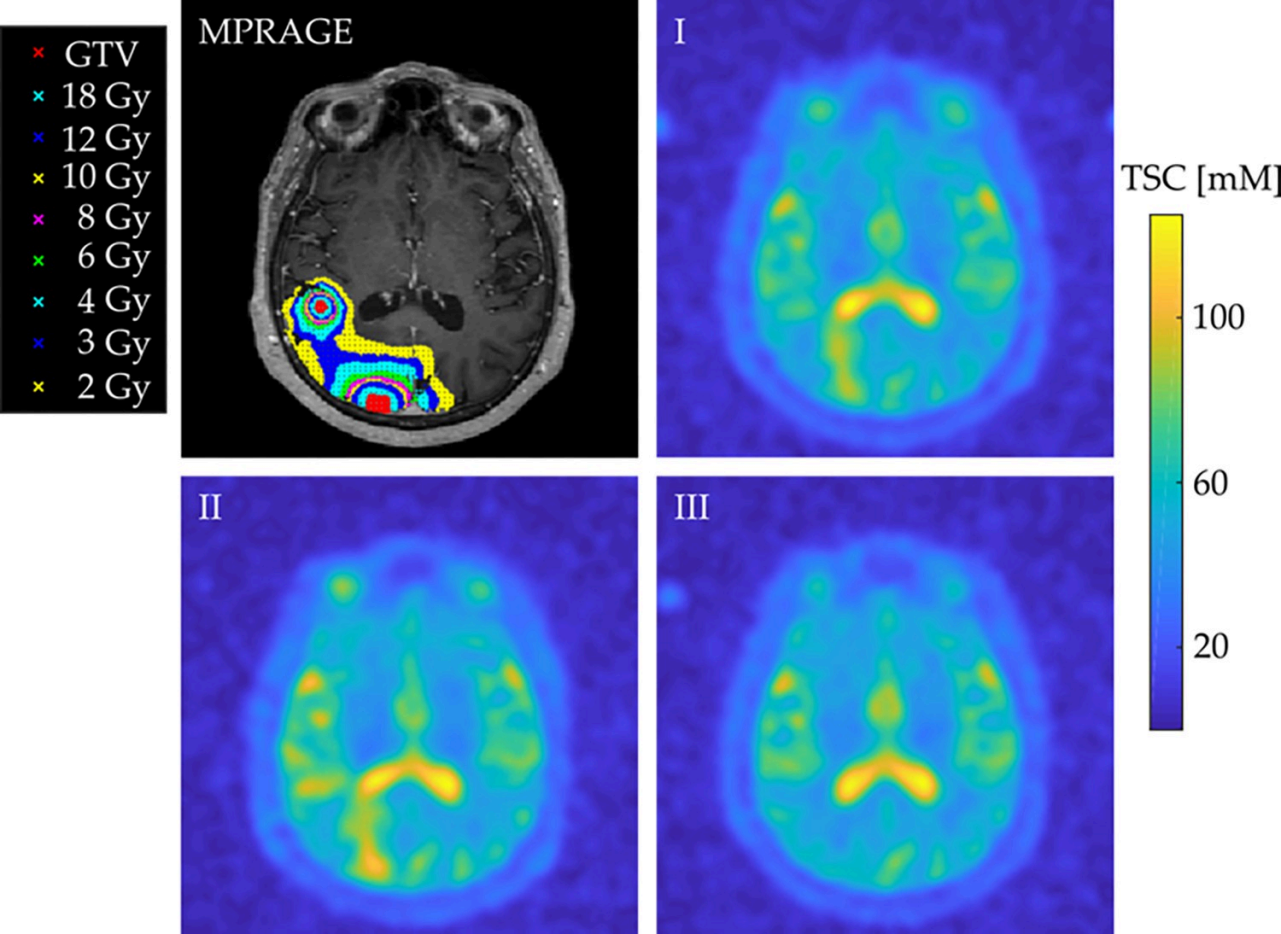
Co-registered MPRAGE and ^23^Na. One representative transverse slice of one patient’s MPRAGE and the co-registered ^23^Na at all three measurements: scan (I) (t = SRS– 2 days), scan (II) (t = SRS + 5 days), and scan (III) (t = SRS + 40 days). The isodose areas and the two GTV are being visualized within the MPRAGE as color-coded regions.

One representative transverse slice of the MPRAGE and of the three ^23^Na MRI of one patient with the GTV, and the isodose line regions being visualized within the MPRAGE are shown in Fig 1.

The mean TSC within all Gross Tumor Volumes (GTV), Healthy ROIs (HR), and the isodose areas of D = 2, 3, 4, 6, 8, 10, 12, 18 at all three scans (I, II, and III) are listed in Table 2.

**Table 2:** Mean TSC within GTV, HR, and isodose areas at all three measurements as mean ± SD. Statistical significance is indicated by ‘*’. (BM: brain metastases).

Region	Scan (I) (TSC in mM)	Scan (II) (TSC in mM)	Compared to (I) (TSC ↑: higher/↓: lower)	Scan (III) (TSC in mM)	Compared to (I) (TSC ↑: higher/↓: lower)	Compared to (II) (TSC ↑: higher/↓: lower)
**GTV (stable BM)**	63 ± 9	71 ± 7		57 ±8		
**GTV (progr. BM: sarcoma)**	50	53		71		
**GTV (progr. BM: mammary)**	48	53		50		
**HR (stable BM)**	45 ± 6	45 ± 5		44 ± 5		
**Isodose Areas (stable BM)**						
**18 Gy**	61 ± 10	67 ± 9	**↑** p = 0.0132*	54 ± 7	**↓** p = 0.0257*	**↓** p = 0.0079*
**12 Gy**	60 ± 10	65 ± 9	**↑** p = 0.0202*	52 ± 6	**↓** p = 0.0180*	**↓** p = 0.0068*
**10 Gy**	59 ± 9	63 ± 9	**↑** p = 0.0352*	51 ± 6	**↓** p = 0.0134*	**↓** p = 0.0062*
**8 Gy**	59 ± 9	63 ± 10	**↑** p = 0.0364*	51 ± 6	**↓** p = 0.0092*	**↓** p = 0.0058*
**6 Gy**	58 ± 8	61 ± 10	↑ p = 0.05	51 ± 6	**↓** p = 0.0081*	**↓** p = 0.0089*
**4 Gy**	56 ± 7	59 ± 9	↑ p = 0.07	50 ± 6	**↓** p = 0.0156*	**↓** p = 0.0140*
**3 Gy**	54 ± 6	56 ± 8	↑ p = 0.18	50 ± 5	**↓** p = 0.0032*	**↓** p = 0.0161*
**2 Gy**	53 ± 5	54 ± 7	↑ p = 0.27	50 ± 6	↓ p = 0.06	↓ p = 0.13

Mean TSC in all HR was 45 ± 5 mM. 45 ± 5 mM, and 44 ± 5 mM at scan (I), (II), (III), respectively. There was no statistically significant difference between any of the three scans (all p > 0.7).

Mean TSC in HR was significantly lower than mean TSC in GTV, at all three scans (all p<0.0001). Mean intra-patient TSC differences in the HR was 2 ± 2 mM between all three scans.

In all evaluated GTV, at baseline, scan (I), mean TSC was 61 ± 10 mM, which evolved to 68 ± 9 mM (+ 11%) at scan (II) and 58 ± 9 mM (- 5% compared to baseline) at scan (III). Differences between scan (I) and scan (III) were not statistically significant (p = 0.27) whereas differences between scan (II) and both other scans were statistically significant (p = 0.0076 and p = 0.0214, respectively).

For the GTV of five brain metastases deriving from non-small cell lung carcinoma (NSCLC), mean TSC was 70 ± 5 mM at scan (I), 76 ± 6 mM at scan (II), and 63 ± 6 mM at scan (III). The GTV of the three brain metastases stemming from mammary carcinoma showed a mean TSC of 54 ± 6 mM at scan (I), 58 ± 4 mM at scan (II), and 48 ± 5 mM at scan (III).

The two progressive brain metastases showed mean TSC values of 50 ± 3 mM (primary tumor: soft tissue sarcoma) and 48 ± 2 mM (primary tumor: mammary carcinoma) at scan (I), 53 ± 5 mM and 53 ± 3 mM at scan (II), and 71 ± 5 mM and 50 ± 3 mM at scan (III), respectively.

**Fig 2:**
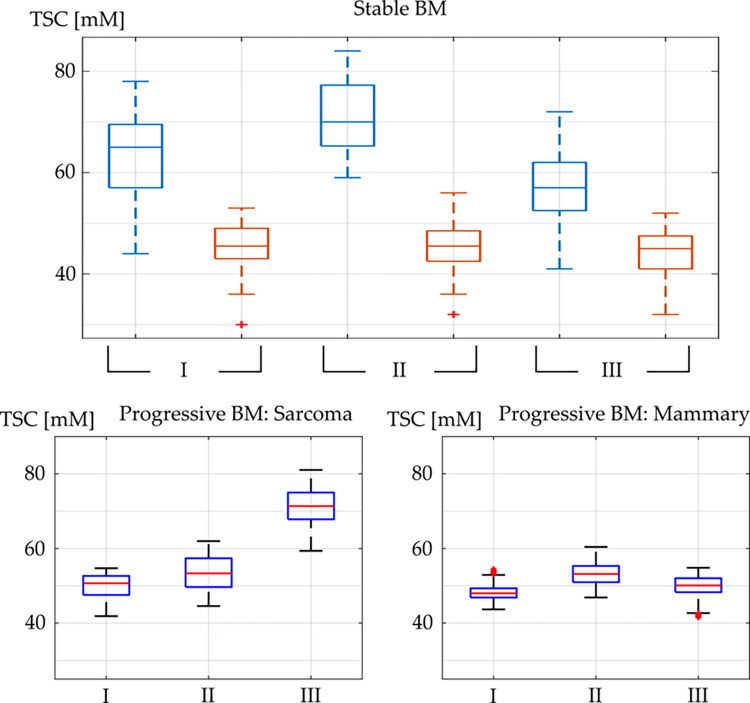
Mean TSC within the GTV. Boxplot of the mean TSC within the GTV (blue), and the HR (red) at all three measurements: scan (I) (t = SRS– 2 days), scan (II) (t = SRS + 5 days), and scan (III) (t = SRS + 40 days). The middle line in the box depicts the median value and the boxes’ top and bottom edges the 25th and 75th percentiles of the data, respectively. The whiskers extend to the most extreme data points not considering outliers which are depicted by “+”. The top figure depicts TSC evolution for stable brain metastases and the two bottom figures depict the TSC evolution of the two progressive brain metastases (left: soft tissue sarcoma, right: mammary carcinoma). (BM: brain metastases).

Mean TSC in GTV and HR at all three scans are depicted as boxplots of the stable brain metastases and of the progressive brain metastases in Fig 2.

The mean TSC within the isodose areas of D = 2, 3, 4, 6, 8, 10, 12, 18 Gy was lower at baseline, scan (I), compared to measurement at scan (II) for all isodose areas and differences were significant for isodose areas D = 18 to 8 Gy but not for isodose areas D = 6 to 2 Gy. Comparing mean TSC within the isodose areas between scan (I) and scan (III), TSC was higher at baseline within all isodose areas and differences were significant for D = 18 to 3 Gy but not for D = 2 Gy. Comparing mean TSC within the isodose areas between measurements at scan (II) and (III), TSC was higher at scan (II) for all isodose areas and differences were also significant for D = 18 to 3 Gy but not for D = 2 Gy. The respective tendencies and corresponding p-values are given in Table 2.

**Fig 3:**
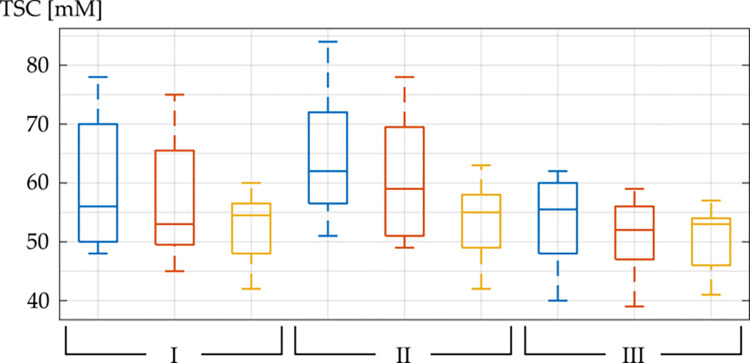
TSC of the peritumoral tissue. Boxplot of the mean TSC of all stable brain metastases within the isodose areas D = 18 Gy (blue), D = 10 Gy (red) and D = 2 Gy (yellow) at all three measurements: scan (I) (t = SRS– 2 days), scan (II) (t = SRS + 5 days), and scan (III) (t = SRS + 40 days). The middle line in the box depicts the median value and the boxes’ top and bottom edges represent the 25th and 75th percentiles of the data, respectively. The whiskers extend to the most extreme data points.

Fig 3 depicts boxplots of the mean TSC within the isodose line D = 2, 10, and 18 Gy at all three measurements as boxplots.

**Fig 4:**
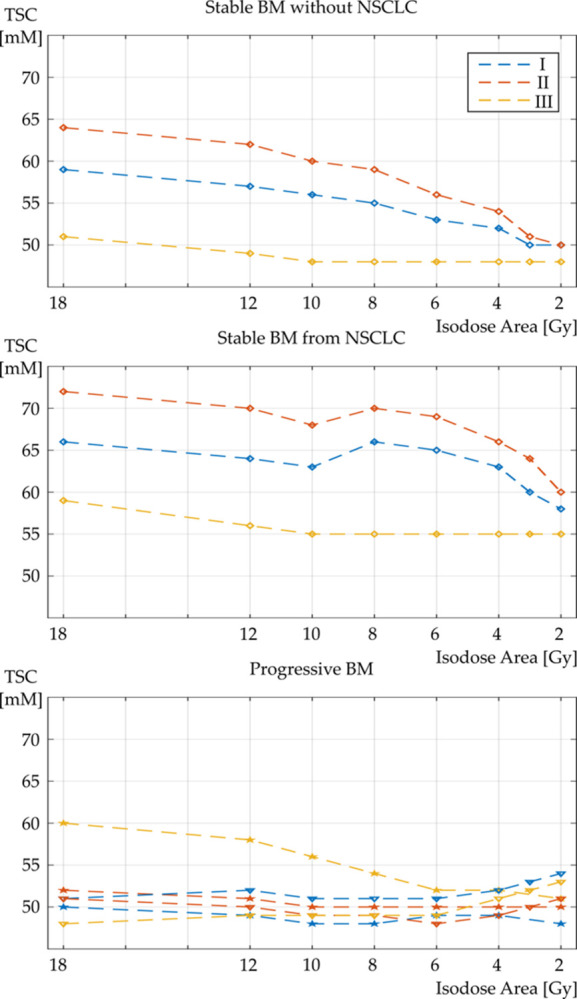
TSC in the peritumoral tissue of stable and progressive brain metastases. Plot of the mean TSC over the observed isodose areas at all three measurements: scan (I) in blue (t = SRS– 2 days), scan (II) in red (t = SRS + 5 days), and scan (III) in yellow (t = SRS + 40 days). The figure shows the TSC evolution separately for patients with stable brain metastases either from NSCLC or from any other primary. The bottom plot shows the TSC evolution of both patients with progressive brain metastases. Here, the patient with a BM from soft tissue sarcoma is depicted with triangles and the patient with a BM from mammary carcinoma is depicted with pentagrams. (BM: brain metastases).

Mean TSC evolution within the isodose areas and their ratio between each other at all three scans (I, II, and III) are depicted in Fig 4.

No significant differences between patients treated with frame fixation compared to those treated with mask fixation were detected in baseline TSC or TSC evolution within the GTV or the isodose areas.

The Pearson correlation test showed a significant, positive correlation between the radiation dose D and the mean TSC within the corresponding isodose area at all three scans. Correlation between D and mean TSC was r = 0.92 (p = 0.0011) at baseline scan (I), r = 0.96 (p = 0.0001) 5 days after SRS at scan (II), and r = 0.86 (p = 0.0061) 40 days after SRS at scan (III).

## Discussion

The aim of our study was to evaluate the feasibility of longitudinal ^23^Na MRI to reveal early changes of peritumoral TSC in addition to changes of TSC in brain metastases after SRS. To our knowledge, this is the first prospective investigation with ^23^Na MRI to evaluate radiation response in the peritumoral tissue of brain metastases. Our findings demonstrate changes of TSC in the peritumoral tissue following SRS, suggesting that ^23^Na MRI is sensitive to treatment-related effects, not only within brain metastases but also within the peritumoral tissue.

### Altered tissue sodium concentration in brain metastases and peritumoral tissue

TSC in tumors depends on intra- and extracellular sodium concentration and the respective relative volumes. Alterations are believed to originate from accelerated cell division, tumor neovascularization and an increased interstitial space [29–34]. While those compartments affect TSC within brain metastases to various degrees, the peritumoral tissue of untreated brain metastases is primarily subjected to sodium-rich vasogenic edema, elevating TSC in the peritumoral tissue of brain metastases as well as malignant brain tumors [18, 19, 35]. Being caused by leakage of tumor vessels, it is unclear whether the extend of edema volume is more associated with vessel density or vessel disfunction in brain metastases. Yet, vessel density in brain metastases as well as edema volume has been shown to differ depending on the primary histology [33, 36, 37].

### SRS-induced changes of tissue sodium concentration

For comparison, we evaluated TSC in healthy-appearing brain tissue that was in line with normal values (between 30–56 mM) for all patients during all scans [38–40]. A very low TSC difference of 2 ± 2 mM was observed in the healthy-appearing white matter, indicating high quantification stability.

Correlating with previously reported data on brain metastases and malignant brain tumors, mean TSC in GTV was elevated compared to healthy-appearing brain tissue with a further rise in mean TSC shortly after SRS and a subsequent decline below initial levels 40 days after SRS [19, 41]. Reflecting radiation-induced changes, the TSC increase within the GTV shortly after SRS can be purported to the acute intracellular sodium overload accompanying radiation-induced cell damage, characterized by an increased cell membrane permeability as part of apoptosis and necrosis [42]. The drop of TSC below baseline 40 days after SRS can be explained by the onset of tumor shrinkage and a decreased fraction of cancer cells within the GTV as well as antiangiogenic effects of SRS [43, 44].

As demonstrated earlier by our group, mean TSC in the peritumoral tissue was elevated at baseline compared to healthy-appearing brain tissue [19]. In the current study, the peritumoral tissue within close proximity to the GTV showed the highest mean TSC, supporting the idea of peritumoral edema originating from leaky tumor vessels and its influence on TSC of the immediate surroundings in untreated brain metastases.

Shortly after SRS, we found a further, significant increase of mean TSC in the peritumoral tissue with the greatest changes in the isodose areas that had been exposed to higher radiation doses and, thus, also are within close proximity to the GTV. Isodose areas that had received doses of 6 Gy or below did not show a significant change. The peritumoral tissue recovered 40 days after SRS with TSC approaching levels of healthy brain tissue in isodose areas that had received doses of 10 Gy or below.

Regarding the histology of the primary tumors, we noted a difference between the peritumoral tissue surrounding brain metastases stemming from NSCLC (5 of 14 brain metastases) and the peritumoral tissue of brain metastases from other primaries. The peritumoral tissue of brain metastases deriving from NSCLC showed a higher mean TSC throughout all isodose areas before, shortly after, and 40 days after SRS than the peritumoral tissue of the other examined brain metastases and displayed an abrupt decrease of TSC in the isodose areas > 6 Gy at scan (I) and (II).

Very early radiation-induced injury to healthy brain tissue has been histopathologically characterized as endothelial damage with an onset hours after irradiation and (partial) restoration after 4 weeks [45–47]. DWI/PWI-MRI derived measurements have revealed SRS-induced changes in the peritumoral tissue as surrogate for vascular alterations in high dose areas receiving >10 Gy [48, 49]. Subsequently, the constantly low mean TSC in the isodose areas ≤ 10 Gy at scan (III) underlines missing or little initial tissue injury. As endothelial damage and other vascular alterations of brain tissue alone might cause an increase of TSC in the peritumoral tissue early after SRS, a combined role of the aforementioned mechanisms together with an increased tumor-derived edema seems plausible.

Regarding the different characteristic of the peritumoral tissue surrounding brain metastases from NSCLC at scan (I) and (II), previous trials evaluating the discrimination of brain metastases histology by DWI/PWI-MRI of the peritumoral tissue have reported indifferent findings [50, 51]. Here, ^23^Na MRI might provide helpful information, as it has been proposed for classification of glioma, but our preliminary data are not sufficient to be conclusive [41, 52].

### Changes of tissue sodium concentration as an early indicator for response

We noted a further distinction in TSC behavior of the peritumoral tissue surrounding the progredient brain metastases (2 of 14 brain metastases) with homogeneously low TSC in all isodose areas before SRS and a missing surge of TSC shortly after SRS. The missing change of TSC in all isodose areas from scan (I) to scan (II) of the progredient brain metastases represents a substantial difference compared to the peritumoral tissue behavior of the responding brain metastases.

For both progredient brain metastases we recorded a comparable reaction of TSC in GTV with low concentrations at scan (I) and (II). Forty days after SRS, TSC of the peritumoral tissue was increased compared to concentrations before or shortly after SRS for the early progredient brain metastasis (soft tissue sarcoma, previously described by our group), whereas the late progredient brain metastasis (mammary carcinoma) did not exhibit a late increase of TSC, neither in the peritumoral tissue nor within the GTV [19]. The underlying mechanisms of the missing reaction of TSC within GTV or the peritumoral tissue after SRS are yet obscure and might point towards a less vascularized and thus less oxygenated tumor together with a slower tumor metabolism including a decreased mitotic rate, yielding radioresistance and a lower sodium signal.

Taken together, TSC characteristics within the GTV and TSC behaviour in the peritumoral tissue after SRS might have already indicated the unfavourable tumor response as early as 5 days post-SRS. Early response prediction at 1 month after treatment utilizing DWI/PWI-MRI has been described for a larger cohort with 70 brain metastases, but with whole brain radiotherapy or SRS as therapy option [53]. Pilot studies examining very early response prediction with DWI/PWI-MRI or chemical exchange saturation transfer (CEST)-MRI at 3 or 7 days post-SRS, respectively, have been conducted with encouraging results [54, 55]. Lastly, emerging computational prediction methods such as radiomics models or convolutional neural networks benefit from additional information that could be provided by ^23^Na MRI [56, 57]. This could help to identify patients who will ultimately fail SRS and allow for timelier adjustment in treatment approach.

### Specifics and limitations

The here presented study must be considered a prospective feasibility study with a small sample size. The data is preliminary and warrants a pilot study with a greater sample size.

We chose a rigid volume model for the GTV and the isodose areas which did not account for volume shifts at scan (II) or (III). Early changes in size of brain metastases after SRS have been shown, but subsequent estimation of an early mass effect on the peritumoral tissue might further increase imprecision [43].

In the future, it would be of high interest to correlate the results obtained from TSC measurements in the peritumoral tissue with additional methods for the assessment of tumor progression, such as DWI or PWI measurements [12–14]. Further limitations of this study include the low spatial resolution of the 3D ^23^Na MR images derived from a sodium-weighted sequence, which did not allow for analysis of brain metastases with a volume of less than 64 mm^3^ and which implies uncertainties about the precision of isodose areas and their TSC quantification due to partial volumes effects as common problem with ^23^Na MRI. However, we addressed this issue by evaluating isodose areas with sufficient distance towards each other, as it is visualised in Fig 1. Distinction between intra- and extracellular TSC, in combination with ultra-high field MRI could further enhance information obtained with ^23^Na MRI in future trials [58].

## Conclusion

Early TSC assessment within brain metastases and the peritumoral tissue after SRS with ^23^Na MRI is feasible. Future investigations are needed to elucidate the reasons for radiation-induced changes in TSC and their clinical value for response prediction.
